# Uninterruptible Power Supply Improves Precision and External Validity of Telomere Length Measurement *via* qPCR

**DOI:** 10.1017/exp.2020.58

**Published:** 2020-11-16

**Authors:** Waylon J. Hastings, Dan T.A. Eisenberg, Idan Shalev

**Affiliations:** 1 Department of Biobehavioral Health, The Pennsylvania State University, University Park PA, USA; 2 Department of Anthropology, University of Washington, Seattle, WA, USA

**Keywords:** telomere length, qPCR, assay precision, power supply

## Abstract

Technical challenges associated with telomere length (TL) measurements have prompted concerns regarding their utility as a biomarker of aging. Several factors influence TL assessment *via* qPCR, the most common measurement method in epidemiological studies, including storage conditions and DNA extraction method. Here, we tested the impact of power supply during the qPCR assay. Momentary fluctuations in power can affect the functioning of high-performance electronics, including real-time thermocyclers. We investigated if mitigating these fluctuations by using an uninterruptible power supply (UPS) influenced TL assessment *via* qPCR. Samples run with a UPS had significantly lower standard deviation (*p* < 0.001) and coefficient of variation (*p* < 0.001) across technical replicates than those run without a UPS. UPS usage also improved exponential amplification efficiency at the replicate, sample, and plate levels. Together these improvements translated to increased performance across metrics of external validity including correlation with age, within-person correlation across tissues, and correlation between parents and offspring.

## 1. Introduction

Telomeres, the repetitive nucleoprotein regions at chromosome ends, are hallmarks of biological aging (Lopez-Otin et al., 2013). Large population studies have associated shorter telomere length (TL) with a range of risk factors that predict health problems and shorter life expectancy (Wang et al., 2018). Even so, technical challenges with TL measurement have led to questions regarding their utility as a biomarker of aging (*e.g*. Hastings et al., 2017).

The most common approach to quantify TL in epidemiological studies is quantitative-PCR (qPCR), which expresses telomeric content relative to a single copy gene (Cawthon, 2002). In addition to concerns of being less precise than measures generated by Southern Blot (Aubert et al., 2012), TL measurement *via* qPCR is subject to influence by several pre-analytical factors including DNA extraction method (Cunningham et al., 2013), sample storage conditions (Dagnall et al., 2017), and analytical factors such as PCR mastermix (Jiménez & Forero, 2018) and well position on plate-based thermocyclers (Eisenberg et al., 2015). However, whether power supply during the qPCR assay influences TL measurement has remained unconsidered.

Momentary fluctuations in power can affect the functioning of high-performance electronics, including real-time thermocyclers. These fluctuations can be mitigated by using an uninterruptible power supply (UPS), an apparatus capable of supplying constant, uninterrupted voltage (Aamir et al., 2016). The current study investigated if UPS usage influenced TL assessment *via* qPCR across a range of technical and external validity metrics.

## 2. Methods

### 2.1. DNA Extraction and Telomere Length Assessment

Whole blood and buccal epithelial cells were collected from 26 grandmothers (age 52.6–72.2), 106 mothers (age 29.1–43.6) and 126 children (45.4% male; age 0.5–24.9). DNA for TL analyses was extracted from buffy coat (N = 94; 12 grandmothers, 79 mothers, & 3 children) and buccal epithelial cells (N = 271; 26 grandmothers, 116 mothers, & 129 children; dataset included multiple time points for 10 mothers and 3 children) using QIAamp DNA Mini Kits (Qiagen, Germany). DNA purity and quality was assessed using 260/230 and 260/280 ratios, but no exclusionary criteria was imposed prior to assays. DNA was stored at −80°C until TL analysis. All TL assays were performed by WJH on a Qiagen Rotor-Gene Q thermocycler, using a qPCR protocol adapted from Cawthon (2002). Each telomere assay comprised two qPCR runs, one run quantifying telomere content (T) and a second run quantifying genome copy number (S) using the single copy gene *36B4*. Detailed descriptions of sample handling and processing, as well as details regarding qPCR assay and quality control are summarized in the supplemental material in accordance with guidelines recommended by the Telomere Research Network (https://trn.tulane.edu/wp-content/uploads/sites/445/2020/08/TRN-Reporting-Guidelines-updated.pdf). The same DNA aliquot was used for T and S runs. Each run hosted triplicate reactions of 22 samples, 5 standards, and 6 positive controls on 100 well disks.

Telomere length was quantified as the T/S ratio, which was calculated as $T/S={\left(\frac{{E_{T}}^{{\text{Ct}}_{T}}}{{E_{S}}^{{\text{Ct}}_{S}}}\right)}^{-1}$, where E_T/S_ is the efficiency of exponential amplification for the telomere or single copy gene respectively, and Ct_T/S_ is the cycle at which a given replicate targeting telomeric content or the single copy gene reaches the critical threshold of fluorescence detection. A serial dilution of five standards were used to identify a critical threshold of detection for extraction of Ct_T/S_ values. Estimates of amplification efficiency at the replicate, sample, and plate levels used data generated from LinRegPCR (Ramakers et al., 2003). T/S ratios were calculated using plate-level efficiencies, which have been shown to decrease bias and variability in qPCR data (Ruijter et al., 2009).

### 2.2. Sample Overview and Statistical Analyses

**Figure 1.**
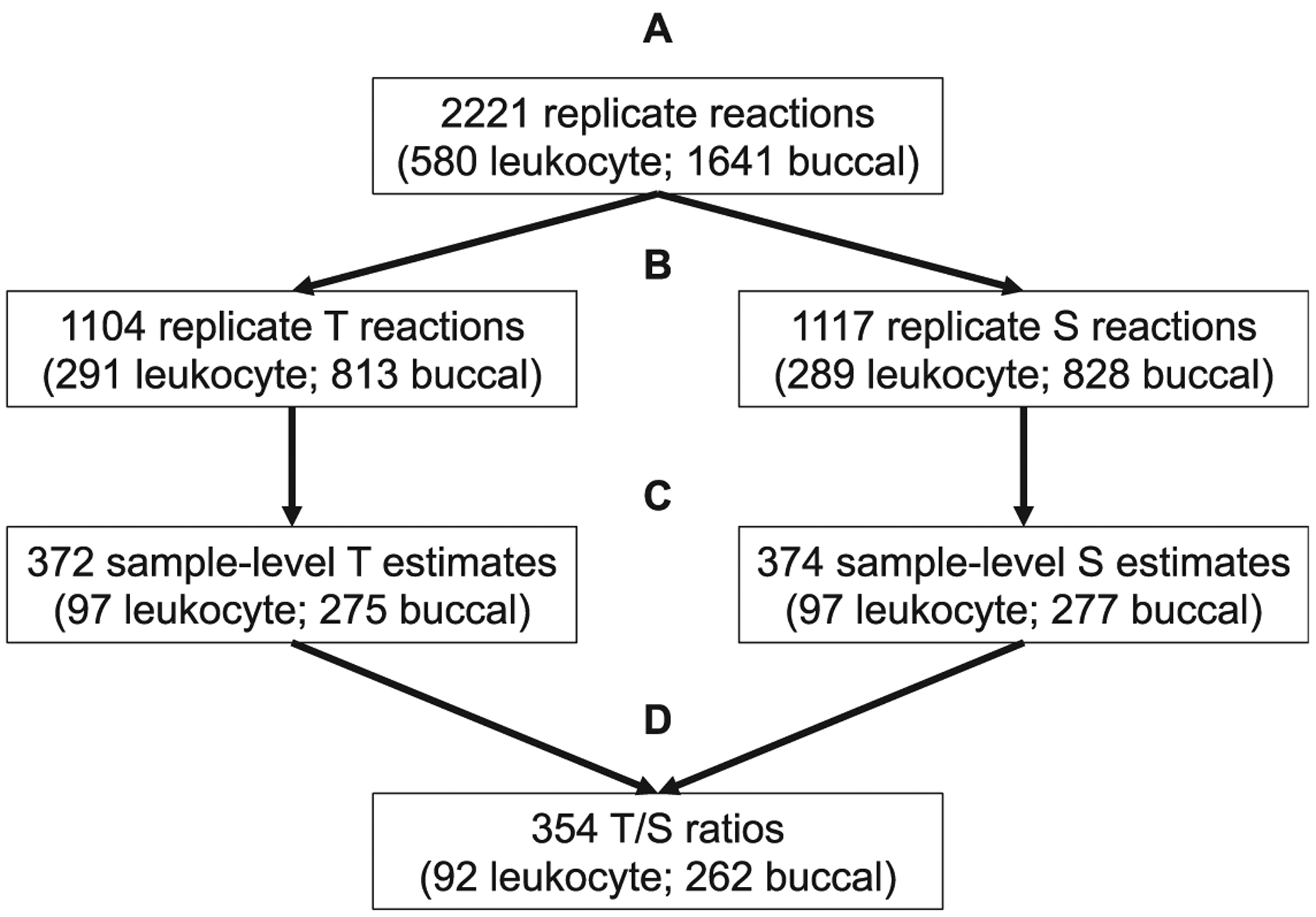
Sample flow and subsets used for analyses. **1A**. 2,221 replicate reactions comprising the full sample. **1B**. Replicate reactions were distinguished by amplification target and analyzed separately due to concerns in reaction chemistry. Replicate reactions were used in analyses of replicate level efficiencies as a function of UPS utilization. **1C**. Technical replicates were clustered by sample ID for analyses of standard deviation and coefficient of variation across replicate level efficiencies, replicate T-estimates, and replicate S-estimates. Differences in sample level efficiencies, calculated as the average efficiency across replicates, were also conducted within this subsample. The two additional data points for single copy gene data correspond to the two telomere samples that did not amplify as described in main text. **1D**. Calculated T-estimates and S-estimates were used to calculate T/S values for 363 samples. Original T/S values for the 9 samples that were rerun were not included in analyses of T/S ratio data. Neither were the 9 T/S values marked as outliers, bringing the final sample size for external validity correlates to 354.

The present work summarizes data generated from TL assessments of 2,221 replicate reactions across 34 qPCR runs (17 T & 17 S) as part of a larger investigation into intergenerational transmission of trauma, as previously reported (Etzel et al., 2020). Sample-level analyses used the standard deviation and coefficient of variation across replicate Ct_T/S_ values, natural log transformed T estimates ($\text{Ln}\left[{E_{T}}^{{\text{Ct}}_{T}}\right]$), and natural log transformed S estimates ($\text{Ln}\left[{E_{S}}^{{\text{Ct}}_{S}}\right]$). Due to potential differences in reaction chemistry, telomere and single copy gene reactions were analyzed independently. A full break down of sample flow and subsets used in each analysis is provided in Figure 1. Results described in the main text represent combined analyses of leukocyte and buccal samples. Independent analyses within each tissue are reported in Supplemental Tables S1–S5. Two telomere samples failing to reach the threshold of detection were removed from analyses. A UPS (Back-UPS Pro 700; APC) was utilized on approximately half of the runs (N = 18; 9 T & 9 S). All samples which had T run with the UPS also had their corresponding S reaction also run with the UPS. The runs utilizing the UPS were situated within the middle of the assays (*i.e*., 5 T runs and 5 S runs without the UPS followed by 9 T runs and 9 S runs using the UPS followed by 4 T runs and 4 S runs without the UPS). Differences in group means were assessed using t-tests. Homogeneity of variances between reactions assessed with and without the UPS was tested using Levene’s test. In instances where group variances were significantly different, the Welch t test was conducted in lieu of the Student’s t test (Welch, 1947).

To better understand how UPS usage would influence the findings derived from telomere length data, we compared how samples assessed on runs with and without a UPS varied in their correlation between T/S ratio and external validity metrics including age, across-tissue within person, and among parents and offspring (Eisenberg, 2016). Differences in T/S ratio correlation coefficients were evaluated based on overlap of 83.4% confidence intervals (Knol et al., 2011). Samples with T/S ratios more than 3 standard deviations above the mean were marked as outliers and removed from analyses (N_(+)UPS_ = 5; N_(−)UPS_ = 4). Statistical analyses were conducted with IBM SPSS Statistics 26. Sample size estimates for reported power calculations were performed in Stata 15.1 using the ‘power onecorrelation’ command with power = 0.80 and α = 0.05, and effect size equal to the observed correlation coefficient.

## 3. Results

**Figure 2.**
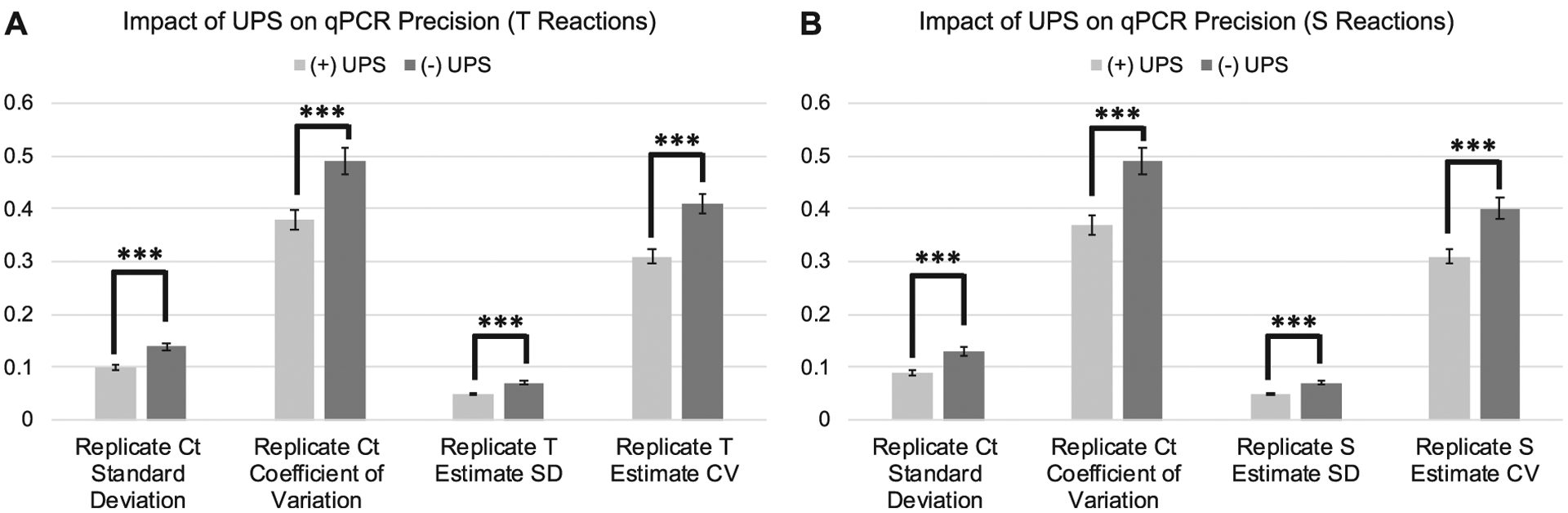
Differences in assay precision as a function of UPS usage delineated by PCR amplification target. **2A**: Average standard deviation and coefficient of variation across replicate Ct_T_ values and T estimates for samples assessed with (N = 196) and without (N = 176) the use of a UPS. **2B**: Average standard deviation and coefficient of variation across replicate Ct_S_ values and S estimates for samples assessed with (N = 198) and without (N = 176) the use of a UPS. Error bars reflect standard error of the mean. SD = Standard Deviation. CV=Coefficient of Variation. ***p < 0.001.

**Figure 3.**
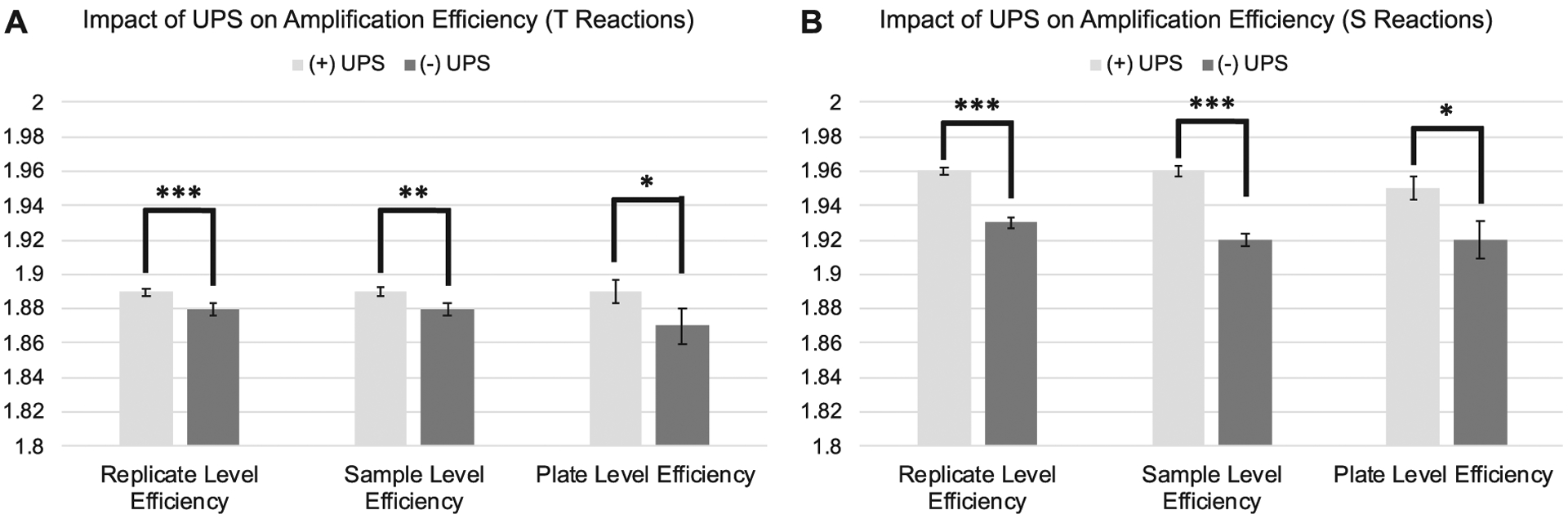
Differences in amplification efficiency as a function of UPS usage delineated by PCR amplification target. **3A**: Average replicate, sample, and plate-level efficiencies for telomere reactions assessed with and without the use of a UPS. **3B**: Average replicate, sample, and plate-level efficiencies for single copy gene reactions assessed with and without the use of a UPS. Efficiencies derived from LinRegPCR. Error bars reflect standard error of the mean. SD = Standard Deviation. CV=Coefficient of Variation. ***p < 0.001; **p < 0.01; *p < 0.05.

The standard deviation and coefficient of variation were significantly lower across replicate Ct_T_ values and natural log transformed T estimates for samples assessed on runs utilizing a UPS relative to those run without a UPS (Figure 2A;Table 1:). Estimates of amplification efficiency at the replicate, sample, and plate level were also significantly improved on runs using a UPS, situating them closer to desired population doubling (Figure 3A; Table 1:). Similar patterns were observed for reactions targeting the single copy gene (Figure 2B;Figure 3B;Table 2:).

UPS status improved all metrics assessing the external validity of T/S ratios (Table 3:). Within-person, cross-tissue correlations were significantly higher for samples assessed with a UPS relative to those without. The correlation between age and T/S ratios and between parent and offspring T/S ratios were also improved for samples assessed with a UPS, but not to a significant extent. Similar patterns were observed when leukocyte and buccal samples were analyzed independently (Supplemental Table S5).

To explore power gains yielded from using the UPS, we compared the sample sizes needed to distinguish UPS *versus* no-UPS TL external validity correlates as significantly different from zero (α = 0.05, power = 0.80.) For example, to detect the correlation of TL across tissues without a UPS (r = 0.62) requires a sample size of 18, while a sample of 7 is required to detect to detect with a UPS (r = 0.92). This equates to being able to detect a significant effect with a 61% smaller sample size. Using the same procedure for age and parent-offspring correlations yields estimates of 25% and 8% smaller samples, for an average ability to detect an effect with a 31% smaller sample.

## 4. Conclusions

TL assessment *via* qPCR is subject to bias from a host of analytical and pre-analytical factors (reviewed in Lin et al., 2019), leading some to challenge the utility of telomeres as a biomarker of aging (Boonekamp et al., 2013). Nevertheless, TL measurement *via* qPCR remains widely used in epidemiological research. Thus, elucidating measurement practices which enhance reproducibility and precision is of great interest.

Our results demonstrate substantial improvements to qPCR assay precision and measures of external validity through the utilization of an uninterruptible power supply. Further, findings suggest utilization of a UPS increases power in a manner equivalent to a 31% increase in sample size, although the degree of such improvement may differ with the baseline electric power quality and type of thermocycler employed. We frame our findings in the context of literature on TL assessment given the aim of the assays comprising our sample. However, the results are likely applicable to qPCR more broadly, and demonstrate the importance of considering power supply when conducting biological assays that rely on high performance electronics.

## Supplementary Material

Supp material

Supp material 2

## Figures and Tables

**Table 1.** Comparing Features of T-Estimates by UPS Status

	(−UPS)	(+) UPS	Test Statistic	*p*-value
Standard Deviation Across Replicate Ct Values	0.14 (0.09)	0.10 (0.07)	t_322.038_ = 4.555	*p* < 0.001
CV Across Replicate Ct Values	0.49 (0.32)	0.38 (0.25)	t_330.442_ = 3.829	*p* < 0.001
Standard Deviation Across Replicate Natural Log Transformed T-Estimates	0.07 (0.04)	0.05 (0.03)	t_329.618_ = 4.294	*p* < 0.001
CV Across Replicate Natural Log Transformed T-Estimates	0.41 (0.26)	0.31 (0.20)	t_331.142_ = 3.909	*p* < 0.001
Replicate Level Efficiency	1.88 (0.08)	1.89 (0.05)	t_921.119_ = −3.571	*p* < 0.001
Standard Deviation Across Replicate Level Efficiencies	0.05 (0.03)	0.04 (0.02)	t_293.912_ = 5.979	*p* < 0.001
Coefficient of Variation Across Replicate Level Efficiencies	2.79 (1.51)	1.88 (0.97)	t_292.945_ = 6.131	*p* < 0.001
Sample Level Efficiency	1.88 (0.05)	1.89 (0.04)	t_311.781_ = −2.963	*p* = 0.003
Plate Level Efficiency	1.87 (0.03)	1.89 (0.02)	t_15_ = −2.430	*p* = 0.028

* Test statistics reported from independent samples t-test. Values reported are Mean (Standard Deviation). CV = coefficient of variation.

**Table 2.** Comparing Features of S-Estimates by UPS Status

	(−UPS)	(+) UPS	Test Statistic	*p*-value
Standard Deviation Across Replicate Ct Values	0.13 (0.09)	0.09 (0.06)	t_321.314_ = 4.571	*p* < 0.001
CV Across Replicate Ct Values	0.49 (0.33)	0.37 (0.26)	t_332.150_ = 3.804	*p* < 0.001
Standard Deviation Across Replicate Natural Log Transformed S-Estimates	0.07 (0.05)	0.05 (0.03)	t_324.946_ = 4.267	*p* < 0.001
CV Across Replicate Natural Log Transformed S-Estimates	0.40 (0.27)	0.31 (0.21)	t_332.144_ = 3.818	*p* < 0.001
Replicate Level Efficiency	1.93 (0.08)	1.96 (0.06)	t_976.353_ = −7.431	*p* < 0.001
Standard Deviation Across Replicate Level Efficiencies	0.05 (0.03)	0.04 (0.02)	t_318.043_ = 4.672	*p* < 0.001
Coefficient of Variation Across Replicate Level Efficiencies	2.57 (1.37)	1.94 (1.01)	t_318.327_ = 4.955	*p* < 0.001
Sample Level Efficiency	1.92 (0.05)	1.96 (0.04)	t_328.426_ = −6.365	*p* < 0.001
Plate Level Efficiency	1.92 (0.03)	1.95 (0.02)	t_15_ = −2.841	*p* = 0.012

* Test statistics reported from independent samples t-test. Values reported are Mean (Standard Deviation). CV = coefficient of variation.

**Table 3.** Comparing Metrics of External Validity by UPS Status

Leukocyte-Buccal Correlation of Plate-Level T/S Ratios		
	r (*p*-value)	83.4% CI
(−) UPS	0.62 (<0.001)	[0.47, 0.74]
(+) UPS	0.92 (<0.001)	[0.88, 0.95]
*Correlations controlled for sex and age		
Correlation Between Age and Plate-Level T/S Ratios		
	r (*p*-value)	83.4% CI
(−) UPS	−0.13 (0.085)	[−0.23, −0.02]
(+) UPS	−0.15 (0.048)	[−0.26, −0.05]
*Correlations controlled sex and tissue (leukocyte/buccal)		
Parent-Offspring Correlation of Plate-Level T/S Ratios		
	r (*p*-value)	83.4% CI
(−) UPS	0.74 (<0.001)	[0.65, 0.80]
(+) UPS	0.78 (<0.001)	[0.70, 0.84]

* Correlations controlled for sex, parental age, offspring age, and tissue (leukocyte/buccal)
