# Supplementary material for: Uninterruptible Power Supply Improves Precision and External Validity of Telomere Length Measurement via qPCR
Source: Exp Results. Author manuscript; Available in PMC 2021 Mar 12. (PMC7954403; doi:10.1017/exp.2020.58)
Supplement: Supp material [file NIHMS1646967-supplement-Supp_material.docx]

**SUPPLEMENTARY MATERIAL**

| **Supplemental Table S1: Comparing Features of T-Estimates by UPS Status (Leukocyte Only)** | | | | |
| --- | --- | --- | --- | --- |
|  | (-UPS) | (+) UPS | Test Statistic | P-value |
| Standard Deviation Across Replicate Ct Values | 0.14 (0.07) | 0.11 (0.07) | t_95_=2.564 | p=0.012 |
| CV Across Replicate Ct Values | 0.52 (0.26) | 0.41 (0.26) | t_95_=2.205 | p=0.030 |
| Standard Deviation Across Replicate Natural Log Transformed T-Estimates | 0.07 (0.04) | 0.06 (0.04) | t_95_=2.387 | p=0.019 |
| CV Across Replicate Natural Log Transformed T-Estimates | 0.43 (0.21) | 0.33 (0.21) | t_95_=2.264 | p=0.026 |
| Replicate Level Efficiency | 1.88 (0.07) | 1.89 (0.05) | t_260.202_=-1.001 | p=0.318 |
| Standard Deviation Across Replicate Level Efficiencies | 0.05 (0.03) | 0.03 (0.02) | t_85.171_=3.491 | p=0.001 |
| Coefficient of Variation Across Replicate Level Efficiencies | 2.66 (1.44) | 1.77 (0.98) | t_84.534_=3.550 | p=0.001 |
| Sample Level Efficiency | 1.88 (0.05) | 1.89 (0.03) | t_87.175_=-0.890 | p=0.376 |
| *Test statistics reported from independent samples t-test. Values reported are Mean (Standard Deviation). CV=coefficient of variation | | | | |

| **Supplemental Table S2: Comparing Features of T-Estimates by UPS Status (Buccal Only)** | | | | |
| --- | --- | --- | --- | --- |
|  | (-UPS) | (+) UPS | Test Statistic | P-value |
| Standard Deviation Across Replicate Ct Values | 0.14 (0.10) | 0.10 (0.07) | t_217.985_=3.731 | p<0.001 |
| CV Across Replicate Ct Values | 0.48 (0.34) | 0.37 (0.24) | t_225.704_=3.019 | p=0.002 |
| Standard Deviation Across Replicate Natural Log Transformed T-Estimates | 0.07 (0.05) | 0.05 (0.03) | t_223.634_=3.529 | p=0.001 |
| CV Across Replicate Natural Log Transformed T-Estimates | 0.40 (0.27) | 0.30 (0.20) | t_226.185_=3.149 | p=0.002 |
| Replicate Level Efficiency | 1.87 (0.08) | 1.89 (0.05) | t_655.921_=-3.525 | p<0.001 |
| Standard Deviation Across Replicate Level Efficiencies | 0.05 (0.03) | 0.04 (0.02) | t_206.213_=4.901 | p<0.001 |
| Coefficient of Variation Across Replicate Level Efficiencies | 2.72 (1.54) | 1.92 (0.97) | t_205.929_=5.039 | p<0.001 |
| Sample Level Efficiency | 1.87 (0.05) | 1.89 (0.04) | t_221.474_=-2.871 | p=0.004 |
| *Test statistics reported from independent samples t-test. Values reported are Mean (Standard Deviation). CV=coefficient of variation. | | | | |

| **Supplemental Table S3: Comparing Features of S-Estimates by UPS Status (Leukocyte Only)** | | | | |
| --- | --- | --- | --- | --- |
|  | (-UPS) | (+) UPS | Test Statistic | P-value |
| Standard Deviation Across Replicate Ct Values | 0.13 (0.09) | 0.11 (0.07) | t_95_=1.342 | p=0.183 |
| CV Across Replicate Ct Values | 0.50 (0.36) | 0.44 (0.29) | t_95_=0.931 | p=0.354 |
| Standard Deviation Across Replicate Natural Log Transformed S-Estimates | 0.07 (0.05) | 0.06 (0.04) | t_95_=1.199 | p=0.234 |
| CV Across Replicate Natural Log Transformed S-Estimates | 0.41 (0.29) | 0.36 (0.23) | t_95_=0.972 | p=0.334 |
| Replicate Level Efficiency | 1.93 (0.06) | 1.97 (0.06) | t_287_=-5.813 | p<0.001 |
| Standard Deviation Across Replicate Level Efficiencies | 0.05 (0.03) | 0.04 (0.02) | t_84.012_=1.746 | p=0.085 |
| Coefficient of Variation Across Replicate Level Efficiencies | 2.47 (1.49) | 1.97 (0.99) | t_83.765_=1.961 | p=0.053 |
| Sample Level Efficiency | 1.93 (0.04) | 1.97 (0.04) | t_95_=-5.337 | p<0.001 |
| *Test statistics reported from independent samples t-test. Values reported are Mean (Standard Deviation). CV=coefficient of variation. | | | | |

| **Supplemental Table S4: Comparing Features of S-Estimates by UPS Status (Buccal Only)** | | | | |
| --- | --- | --- | --- | --- |
|  | (-UPS) | (+) UPS | Test Statistic | P-value |
| Standard Deviation Across Replicate Ct Values | 0.13 (0.08) | 0.09 (0.06) | t_288.663_=4.530 | p<0.001 |
| CV Across Replicate Ct Values | 0.49 (0.32) | 0.35 (0.25) | t_236.077_=3.889 | p<0.001 |
| Standard Deviation Across Replicate Natural Log Transformed S-Estimates | 0.07 (0.04) | 0.05 (0.03) | t_231.497_=4.256 | p<0.001 |
| CV Across Replicate Natural Log Transformed S-Estimates | 0.40 (0.26) | 0.29 (0.20) | t_235.958_=3.872 | p<0.001 |
| Replicate Level Efficiency | 1.92 (0.08) | 1.95 (0.06) | t_685.402_=-5.412 | p<0.001 |
| Standard Deviation Across Replicate Level Efficiencies | 0.05 (0.03) | 0.04 (0.02) | t_233.367_=4.441 | p<0.001 |
| Coefficient of Variation Across Replicate Level Efficiencies | 2.60 (1.33) | 1.93 (1.02) | t_233.560_=4.635 | p<0.001 |
| Sample Level Efficiency | 1.92 (0.05) | 1.95 (0.04) | t_223.562_=-4.569 | p<0.001 |
| *Test statistics reported from independent samples t-test. Values reported are Mean (Standard Deviation). CV=coefficient of variation. | | | | |

| **Supplemental Table S5: Correlation Between Age and Plate-Level T/S Ratios by Tissue** | | |
| --- | --- | --- |
| Leukocyte Only | | |
|  | **r (p-value)** | **83.4% CI** |
| **(-) UPS** | -0.13 (0.367) | [-0.33 , 0.07] |
| **(+) UPS** | -0.18 (0.254) | [-0.38 , 0.04] |
| Buccal Only | | |
|  | **r (p-value)** | **83.4% CI** |
| **(-) UPS** | -0.13 (0.140) | [-0.24 , -0.01] |
| **(+) UPS** | -0.16 (0.083) | [-0.28 , -0.03] |
| *Correlations controlling for sex | | |
