# Supplementary material for: Uninterruptible Power Supply Improves Precision and External Validity of Telomere Length Measurement via qPCR
Source: Exp Results. Author manuscript; Available in PMC 2021 Mar 12. (PMC7954403; doi:10.1017/exp.2020.58)
Supplement: Supp material 2 [file NIHMS1646967-supplement-Supp_material_2.docx]

**Telomere Research Network Reporting Guidelines**

| **ITEM** | **DESCRIPTION** |
| --- | --- |
| **Sample Type, Storage, Extraction, and Integrity** | |
| Sample type | DNA samples were extracted from buffy coat isolated from whole blood collected via 10mL EDTA blood tubes (n=92; 12 grandmothers, 77 mothers, & 3 children) and buccal epithelial cells collected using buccal swabs (Isohelix) (262; 26 grandmothers, 113 mothers, & 122 children) |
| Sample storage conditions, including temperature, duration, and buffer | Buffy coat cells were stored at -80°C in a solution consisting of phosphate buffered saline (PBS) pH 7.2+EDTA (2mMol) + bovine serum albumin (BSA; 0.5%) prior to extraction. Buccal swabs were stored dry (no buffer) in snap top tubes at -80 prior to extraction. Duration between sample collection and DNA extraction ranged from 3.3 to 0.2 years with an average of 1.8 years. |
| DNA extraction method | QIAmp DNA Minikit (Qiagen) with no modification from factory guidelines. |
| DNA storage conditions, including freeze-thaw cycles | DNA was stored -80°C in QIAmp DNA Minikit ‘Buffer AE’. On average there were two freeze thaws for DNA samples between extraction and the qPCR assay. The first was to determine DNA concentration using Quant-iT PicoGreen reagent (Qiagen) and the second was to perform a dilution for the qPCR assay. Samples needed to be reassessed on qPCR assays (n=9) were thawed one additional time. Samples were stored for an average of 7.7 months between extraction and the PicoGreen assay and for another 10.5 months between the PicoGreen assay and qPCR assay. |
| Method of documenting DNA quality and integrity | DNA was quantified using Quant-iT PicoGreen Reagent (mean_buccal_=7.83 ng/uL; mean_leukocyte_=16.26ng/uL). DNA purity and quality was assessed using 260/230 and 260/280 ratios for a subset of blood samples (n=73, mean_260/230_=2.54; mean_260/280_=1.82), but no exclusionary criteria was imposed prior to assays. |
| Percentage of samples specifically tested for DNA quality and integrity | 100% |
| **qPCR Assay** | |
| Method (qPCR, MMqPCR, aTL, etc.) | qPCR, wherein each telomere assay comprised two qPCR runs, one run quantifying telomere content (T) and a second run quantifying genome copy number (S) using the single copy gene *36B4.* The two runs (T & S) were always performed on the same day using the same DNA aliquot which was stored at room temperature between runs (~2.5 hours). Each run hosted triplicate reactions of 22 samples, 5 standards, and 6 positive controls on 100 well disks.  qPCR assays were conducted across a period of 2.27 months. This included approximately two weeks of runs without using the UPS (5 T and 5 S), followed by approximately four weeks using the UPS (9 T and 9 S), and another two weeks without the UPS (4 T and 4 S). |
| PCR machine type | Qiagen Rotor-Gene Q using 100 well disks |
| Source of master mix and reagents, and final reaction volume | The final reaction mix for the telomeric DNA contains 1x QuantiTect SYBR Green Master Mix (Qiagen), 0.2U Uracil Glycosylase (Thermo Fisher Scientific), 0.1 u M forward primer, 0.1 u M reverse primer, and 3 ng DNA in a 20 u L reaction. The reaction mix for 36B4 contains 1x QuantiTect SYBR Green Master Mix, 0.2U Uracil Glycosylase, 0.3 u M forward primer, 0.5 u M reverse primer, and 3 ng DNA in a 20 u L reaction. |
| Telomere primer sequences and concentration | Forward Primer: 5'CGGTTTGTTTGGGTTTGGGTTTGGGTTTGGGTTTGGGTT3′  Reverse Primer: 5'GGCTTGCCTTACCCTTACCCTTACCCTTACCCTTACCCT3′ |
| Single copy gene name, primer sequences, and concentration | *36B4* Forward Primer 5'CAGCAAGTGG-GAAGGTGTAATCC3′  *36B4* Reverse Primer 5'CCCATTCTATCATCAACGGGTACAA3′ |
| Full PCR program description including temperature, times, and cycle numbers | 50 °C – 2min  95 °C – 15min  95 °C – 15s followed by 60 °C for 1 min (data acquisition); 45 cycles. |
| PCR efficiency of single copy gene and telomere primers | N/A. Per the aims of this work these varied between conditions according to UPS usage. |
| Source and concentration of control samples and standard curve | 6 positive controls were selected from within the sample such that each plate included control DNA extracted from blood of grandmothers, buccal of grandmothers, blood of mothers, buccal of mothers, and buccal of children. Standards consisted of double stranded oligomers. |
| **Data Analysis** | |
| Mean and standard deviation or median range of telomere lengths | T/S ratio mean (SD) = 0.70 (0.41)  Buccal T/S ratio mean (SD): 0.67 (0.40)  Leukocyte T/S ratio mean (SD): 0.78 (0.44) |
| Number of sample replicates | Each sample was assessed for T and S on a single run with three replicates within the run. If the sample did not pass quality control criteria described below it was run a second time. |
| Level of independence of replicates | Replicates were drawn from the same DNA aliquot (i.e., the same tube). |
| Analytic method, considering replicate measurements, to determine final length | T and S estimates were calculated using the Ct_T/S_ values for individual replicates. T/S ratios were calculated using the average T and average S estimates across replicates. |
| Method of accounting for variation between replicates | When the standard deviation across replicate Ct values was greater than 0.25, replicate Ct values were evaluated based upon their deviation from mean Ct across triplicates. If one replicate deviated from the mean Ct by more than 15% it was considered an outlier and the mean Ct was recalculated using two replicates. This occurred for 12 T replicates and 5 S replicates among samples that were not rerun. In the case where Ct standard deviation for either T or S replicates was still greater than 0.25 after removal of a single outlier, or was greater than 0.25 without a clear outlier defined by the criteria above, the sample was reassessed for both telomere content and genome copy number and subjected to the same quality control evaluation. A total of 9 samples were rerun a second time. |
| Method of accounting for well position effects within plates | The unique rotary design of the Rotor Gene Q is optimized to minimize well position effects. As such no accounting for such effects was performed. |
| Method of accounting for between plate effects | N/A. As the aim of the current work was to understand how UPS influences variability across and within plates no accounting of between plate effects was conducted. |
| % of samples repeated and % of samples failing QC and excluding from further analyses | 9/354 = 2.5% of samples repeated.  2/354 = 0.56% of samples failed QC and excluded from analyses. |
| Acceptable range of PCR efficiency for single copy gene and telomere primers | N/A given the aim of the work included understanding the role of UPS on PCR efficiency. |
| ICCs of samples/study groups to address variability | ICC not calculated because the goal of this study was to compare within laboratory variation within replicates of samples with and without using a UPS. |
| T/S ratio transformed to a z-score prior before comparison across methods/studies | N/A. No comparison across studies was conducted. |
| How samples nested within families were accounted for | Samples collected from different tissues of the same participant, different time points for the same participant (n=11), and samples collected from different generations or siblings within the same family were always assessed on the same qPCR run. |
